# 

**Published:** 1883-08

**Authors:** 


ORIGINAL COMMUNICATIONS:
Extraction of Deciduous Teeth. N. W. Kingsley, D. D. S., .......... 397
A Case of Severe Purulent Inflammation of the Middle Ear, with Restoration of the Drumhead; Consecutive Dentalgia without Caries. Edward S. Peck, M. A., M. D., .................... 401
The Legal and Moral Responsibility of Dentists in the Administration of Nitrous Oxide Gas. Dr. J. Allen Osmun .................... 404
Theory vs. Facts. Dr. A. M. Ross .................... 412
ORIGINAL TRANSLATIONS AND ABSTRACTS:
Dentists' Instruments .................... 415
To Remove Rust .................... 415
A New Method for the Manufacture of Metal Plates .................... 415
Experiments with Various Plastic Filling Materials .................... 417
REPORTS OF SOCIETY MEETINGS:
Central Dental Association of Northern New Jersey .................... 418
EDITORIAL:
Facial Neuralgia .................... 423
University of Buffalo .................... 428
Dental Calendar .................... 429
American Dental Association .................... 429
Another Use for Carbolic Acid .................... 430
Errata .................... 431
NEW APPLIANCES AND MATERIALS:
New Liquid Gas Apparatus and Dental Cabinet .................... 431
Pneumatic Plugger .................... 431
New Laboratory Furnace .................... 432
BIBLIOGRAPHICAL:
Our Book Table .................... 433
An Index to the Practice of Medicine .................... 434
A Treatise on Artificial Crowns; Historical and Descriptive .................... 435
CURRENT NEWS AND OPINION:
The Dental Calendar .................... 436
American Dental Association .................... 440
American Dental Convention .................... 440
Notice to State Boards of Dental Examiners .................... 441
Sixth Annual Meeting of the American Society of Microscopists ...... 441
Blessed America .................... 441
Honor to a Dentist .................... 442
SELECTIONS:
Facial Neuralgia .................... 442
Dropsy of the Antrum—Punctured and Drained—Cure .................... 445
The Effect of Tobacco on Children .................... 447
Removal of a Maxillary Tumor by Means of the Dental Engine ....... 448
Nitrous Oxide in Labor .................... 449
A Tooth at Birth .................... 449
Good Diagnosis .................... 450
Terms of Pupilage .................... 450
The Extension of Vice .................... 450
POPULAR SCIENCE DEPARTMENT:
The Medicaments of Brutes .................... 451
The Gradual Cooling of the Earth .................... 452
Effect of Low Temperatures upon Magnets .................... 452
To Disguise the Taste of Medicines .................... 453

